# Multiple Lineages of Hantaviruses Harbored by the Iberian Mole (*Talpa occidentalis*) in Spain

**DOI:** 10.3390/v15061313

**Published:** 2023-06-02

**Authors:** Se Hun Gu, Marcos Miñarro, Carlos Feliu, Jean-Pierre Hugot, Naomi L. Forrester, Scott C. Weaver, Richard Yanagihara

**Affiliations:** 1 Department of Tropical Medicine, Medical Microbiology and Pharmacology, John A. Burns School of Medicine, University of Hawaii at Manoa, Honolulu, HI 96813, USA; sehun.gu@gmail.com; 2 Department of Horticultural and Forestry Crops, Servicio Regional de Investigación y Desarrollo Agroalimentario (SERIDA), 33300 Villaviciosa, Spain; mminarro@serida.org; 3 Department of Biology, Health and Environment, Faculty of Pharmacy, University of Barcelona, 08028 Barcelona, Spain; cfeliu@ub.edu; 4 Department of Systematics and Evolution, Muséum National d’Histoire Naturelle, 75005 Paris, France; jean-pierre.hugot@mnhn.fr; 5 School of Life Sciences, Keele University, Keele ST5 5BG, UK; n.forrester-soto@keele.ac.uk; 6 Institute for Human Infections and Immunity and World Reference Center for Emerging Viruses and Arboviruses, University of Texas Medical Branch, Galveston, TX 77555, USA; sweaver@utmb.edu

**Keywords:** *Hantaviridae*, hantavirus, talpid, evolution, Spain

## Abstract

The recent detection of both Nova virus (NVAV) and Bruges virus (BRGV) in European moles (*Talpa europaea*) in Belgium and Germany prompted a search for related hantaviruses in the Iberian mole (*Talpa occidentalis*). RNAlater^®^-preserved lung tissue from 106 Iberian moles, collected during January 2011 to June 2014 in Asturias, Spain, were analyzed for hantavirus RNA by nested/hemi-nested RT-PCR. Pairwise alignment and comparison of partial L-segment sequences, detected in 11 Iberian moles from four parishes, indicated the circulation of genetically distinct hantaviruses. Phylogenetic analyses, using maximum-likelihood and Bayesian methods, demonstrated three distinct hantaviruses in Iberian moles: NVAV, BRGV, and a new hantavirus, designated Asturias virus (ASTV). Of the cDNA from seven infected moles processed for next generation sequencing using Illumina HiSeq1500, one produced viable contigs, spanning the S, M and L segments of ASTV. The original view that each hantavirus species is harbored by a single small-mammal host species is now known to be invalid. Host-switching or cross-species transmission events, as well as reassortment, have shaped the complex evolutionary history and phylogeography of hantaviruses such that some hantavirus species are hosted by multiple reservoir species, and conversely, some host species harbor more than one hantavirus species.

## 1. Introduction

Hantavirology dates to the landmark discovery of Hantaan virus as the prototype virus of hemorrhagic fever with renal syndrome (HFRS) in the striped field mouse (*Apodemus agrarius coreae*) [1]. This was followed by the detection of other Hantaan-like viruses in the bank vole (*Myodes glareolus*) [2] and in the brown rat (*Rattus norvegicus*) and black rat (*Rattus rattus*) [3]. In the Americas, hantaviruses burst into the public consciousness when an outbreak of a rapidly progressive, frequently fatal cardiorespiratory disease, now known as hantavirus cardiopulmonary syndrome (HCPS), occurred in the southwestern United States [4,5]. Cases of HCPS, with case-fatality rates exceeding 20%, have since been diagnosed throughout North and South America [6,7].

Hantaviruses possess a single-stranded, negative-sense RNA genome consisting of three segments designated large (L), medium (M) and small (S), which encode a nucleocapsid (N) protein, two envelope glycoproteins (Gn and Gc), and an RNA-dependent RNA polymerase (RdRP), respectively [8,9]. Hantaviruses have been recently reclassified into a newly designated family, *Hantaviridae*, of the order *Bunyavirales* [10,11]. Viruses in the *Mammantavirinae* subfamily are grouped into four genera: *Orthohantavirus*, *Loanvirus*, *Mobatvirus*, and *Thottimvirus*, based on diversity partitioning by hierarchical clustering (DEmARC) analysis of the concatenated amino acid-coding regions of the full-length S and M segments [10,11]. 

During the past 15 years, the discovery of rodent-borne hantaviruses has been eclipsed by the detection of a vast array of genetically distinct hantaviruses in shrews (order Eulipotyphla, family Soricidae) and bats (order Chiroptera, suborders Yangochiroptera and Yinpterochiroptera) of multiple species from widely separated geographic regions in Europe, Asia, Africa and the Americas [12,13,14]. Additionally, in analyzing frozen and RNAlater^®^-preserved tissues from more than 500 moles, belonging to 11 of the approximately 40 extant species (order Eulipotyphla, family Talpidae), five genetically distinct hantaviruses have been described: Asama virus (ASAV) in the Japanese shrew mole (*Urotrichus talpoides*) from Japan [15]; Oxbow virus (OXBV) in the American shrew mole (*Neurotrichus gibbsii*) [16] and Rockport virus (RKPV) in the eastern mole (*Scalopus aquaticus*) [17] from the United States; Nova virus (NVAV) in the European mole (*Talpa europaea*) from Hungary [18], France [19], Poland [20] and Belgium [21]; and Dahonggou Creek virus (DHCV) in the long-tailed mole (*Scaptonyx fusicaudus*) from China [22]. Recently, additional mole-borne hantaviruses have been reported: Bruges virus (BRGV) in the European mole from Belgium, Germany and the United Kingdom [23]; Academ virus (ACDV) in the Siberian mole (*Talpa altaica*) from Russia [24]; and Landiras virus (LDRV) in the Aquitanian mole (*Talpa aquitania*) from France [25]. 

While all hantaviruses harbored by rodents, including those associated with HFRS and HCPS, and nearly all of the genetically distinct hantaviruses recently detected in shrews belong to the genus *Orthohantavirus*, hantaviruses harbored by moles are found in three of the four *Mammantavirinae* genera. That is, ASAV, OXBV, RKPV, BRGV, ACDV and LDRV are orthohantaviruses; DHCV appears to be a thottimvirus; and NVAV is a mobatvirus. NVAV, which shares a common ancestry with several highly divergent bat-borne mobatviruses, is widespread throughout the vast distribution of the European mole, with prevalence exceeding 50% in France, Poland and Belgium, suggesting a long-standing reservoir host–virus relationship [19,20,21]. The recent demonstration that both NVAV and BRGV are hosted by European moles [23] prompted a search for NVAV- and BRGV-related hantaviruses in the Iberian or Spanish mole (*Talpa occidentalis*) (family Talpidae, subfamily Talpinae). Quite unexpectedly, Iberian moles were found to host NVAV, BRGV and a novel, previously unrecognized hantavirus, designated Asturias virus (ASTV). The finding of multiple hantavirus lineages in the Iberian mole serves as another reminder about the complex evolutionary history of hantaviruses. 

## 2. Materials and Methods

### 2.1. Tissue Specimens

Reducing crop damage requires reliable surveillance of pest vole species in apple orchards. As sanctioned by the Spanish Royal Decree from 2008, permission is not required for orchard growers to kill voles and, in fact, they are obligated to kill pest voles, using snap traps (Topcat Andermatt Biocontrol, Switzerland or Supercat, Swinsinno Solutions, Switzerland). In a study to develop sampling methods for estimating the density of montane water voles (*Arvicola scherman*, formerly *A. terrestris*) and Lusitanian pine voles (*Microtus lusitanicus*) in apple orchards in Asturias, conducted during January 2011 to June 2014 [26], apple growers unintentionally trapped Iberian moles, crowned shrews (*Sorex coronatus*), and greater white-toothed shrews (*Crocidura russula*). Instead of discarding the carcasses, apple growers delivered the dead moles, shrews and rodents to us, and we dissected and preserved the lung tissues in RNAlater^®^ Stabilization Reagent (Qiagen, Valencia, CA, USA) for use in this study. 

### 2.2. RNA Extraction and RT-PCR Analysis

Total RNA, extracted from lung tissues, using the PureLink Micro-to-Midi total RNA purification kit (Invitrogen, San Diego, CA, USA), was reverse transcribed into cDNA, using the SuperScript III First-Strand Synthesis Systems (Invitrogen), with an oligonucleotide primer (OSM55F, 5′-TAGTAGTAGACTCC-3′) designed from conserved 5′-ends of the S, M and L segments of hantaviruses [18]. Next, cDNA were screened by nested PCR using oligonucleotide primers directed at a conserved region of the RdRP gene [27]: HAN-L-F1 (5′-ATG-TAYGTBAGTGCWGATGC-3′), and HAN-L-R1 (5′-AACCADTCWGTYC-CRTCATC-3′), then HAN-L-F2 (5′-TGCWGATGCHACAARTGGTC-3′) and HAN-L-R2 (5′-GCRTCRTCWGARTGRTGDGCAA-3′). Hantavirus RNA-positive samples were then analyzed for S- and M-segment sequences: OSM55 and HTN-S6 (5′-AGCTCNGGATCCATNTCATC-3′), followed by Cro-2F (5′-AGYCCNGTNATGRGWGTNRTYGG-3′) and Cro-2R (5′-ANAYTGRTAR-AANGANGAYTTYTT-3′) for the S segment; and OSV697F (5′-GGACCAGGTGCADCTTGTGAAGC-3′) and TM1485R (5′-CCAGCCA-AARCARAATGT-3′), then TM1199F (5′-TAAVTTCAMCAACATGTCT-3′) and TM1485R for the M segment. Nested or hemi-nested PCR was performed in 20-μL reaction mixtures containing 250 μM dNTP, 2.5 mM MgCl_2_, 1 U of LA Taq polymerase (Takara, Shiga, Japan) and 0.25 μM of each primer. Initial denaturation at 94 °C for 2 min was followed by two cycles each of denaturation at 94 °C for 30 s, two-degree step-down annealing from 46 °C to 38 °C for 40 s, and elongation at 72 °C for 1 min, then 30 cycles of denaturation at 94 °C for 30 s, annealing at 42 °C for 40 s, and elongation at 72 °C for 1 min, in a GeneAmp PCR 9700 thermal cycler (Perkin-Elmer, Waltham, MA, USA) [18,20]. PCR products were separated using MobiSpin S-400 spin columns (MoBiTec, Goettingen, Germany), and amplicons were sequenced directly using an ABI Prism 3130 Genetic Analyzer (Applied Biosystems, Foster City, CA, USA) [20]. 

### 2.3. Next-Generation Sequencing

cDNA samples were processed and run on the HiSeq 1500 System (Illumina, Inc., San Diego, CA, USA) at the University of Texas at Galveston Sequencing Core Facility, using the methods described previously [28,29]. Seven hantavirus-positive samples were mulitplexed on a single lane. Reads were sorted and barcodes removed, quality control was applied and the samples were assembled into contigs. Contigs were subjected to basic local alignment search tool (BLAST) analysis to determine their origins. 

### 2.4. Genetic and Phylogenetic Analyses

Pairwise alignment and comparison of nucleotide sequences were performed using Clustal W [30]. Unrooted phylogenetic trees were generated by maximum-likelihood and Bayesian methods, implemented in RAxML Blackbox webserver [31] and MrBayes 3.1 [32], under the best-fit GTR + I + Г model of evolution selected by hierarchical likelihood-ratio test in MrModeltest v2.3 [33] and jModelTest version 0.1 [34]. Two replicate Bayesian Metropolis–Hastings Markov Chain Monte Carlo runs, each comprising six chains of 10 million generations sampled every 100 generations with a burn-in of 25,000 (25%), resulted in 150,000 trees overall. Each genomic segment (S, M and L) was treated separately in phylogenetic analyses. The posterior node probabilities were based on 2 million generations and estimated sample sizes over 100 (implemented in MrBayes).

### 2.5. Host Identification and Phylogeny

To molecularly verify the species of the hantavirus-infected mole hosts, the 1140-nucleotide cytochrome *b* gene of mitochondrial DNA (mtDNA) was amplified by PCR using universal primers (forward, 5′-CGAAGCTTGATATGAAAAACCAT-CGTTG-3′; and reverse, 5′-CTGGTTTACAAGACCAGAGTAAT-3′) [35]. PCR was performed in 50-μL reaction mixtures containing 200 mM dNTP and 1.25 U of LA Taq polymerase (Takara). Cycling conditions consisted of an initial denaturation at 95 °C for 4 min, followed by 40 cycles with denaturation at 94 °C for 1 min, annealing at 57 °C for 1 min, and elongation at 72 °C for 1 min in a GeneAmp PCR9700 thermal cycler. Phylogenetic analysis was performed using the maximum-likelihood method [36], and evolutionary analyses were conducted in MEGA 7 [37].

## 3. Results

### 3.1. Hantavirus RNA Detection

**Figure 1:**
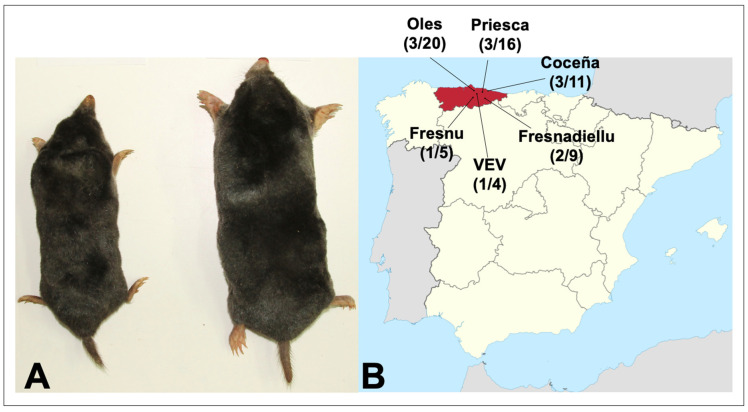
(**A**) Adult Iberian mole (*Talpa occidentalis*) and European mole (*Talpa europaea*), showing the comparatively smaller size of the former species. (**B**) Map showing sites in Asturias in northwestern Spain, where hantavirus-infected Iberian moles and crowned shrews were collected. The number of hantavirus RNA-positive animals and the number tested are shown for each site.

Hantavirus RNA was detected by nested RT-PCR, using L-segment oligonucleotide primers, in 11 of 56 Iberian moles captured in Coceña, Fresnadiellu, Oles and Priesca, four parishes in the principality of Asturias, in northwestern Spain (Figure 1 and Table 1). Additionally, 2 of the 15 crowned shrews, one each in VEV and Fresnu, were positive (Table 1). 

Hantavirus-infected Iberian moles were collected in Coceña (43°28′52.05″ N, 5°14′56.50″ W), Fresnadiellu (43°22′25.70″ N, 5°26′02.60″ W), Oles (43°31′46.39″ N, 5°27′04.55″ W), and Priesca (43°29′06.50″ N, 5°21′39.48″ W), and infected crowned shrews were collected in VEV (Villaviciosa) (43°28′28.40″ N, 5°26′35.00″ W) and Fresnu (43°24′21.25″ N, 5°32′00.08″ W). Hantavirus RNA was not detected in an additional 50 Iberian moles collected during the same period in Aizarnazabal (1); Aritzia (1); BGV (Villaviciosa) (7); Ceceda (1); Deva (1); Hijas (1); La Fajera (5); La Rozada (1); Llata (1); Merón (11); Petritegui-Astigarraga (1); Poreño (6); PT (Villaviciosa) (2); Sta. Marina (1); Tuero (1); Usurbil (1); Vegadali (8), as well as in 76 greater white-toothed shrews, 41 Lusitanian pine voles, and 40 montane water voles.

### 3.2. Sequence Analysis

Based on the analysis of the partial L-segment sequences, BRGV, NVAV and ASTV were identified in six, four and one Iberian moles, respectively (Table 2). Despite multiple attempts, we were unable to obtain S- and M-segment sequences of NVAV and BRGV from Iberian moles. Interestingly. two infected Iberian moles, captured in the same gallery on two consecutive days (TO11.06.14.02 and TO11.06.15.01) in Coceña, harbored different hantaviruses: NVAV 3873 and BRGV 3879.

Pairwise alignment and comparison of the full-length S and the partial M and L segments, amplified from an Iberian mole trapped in Coceña on 14 June 2011, showed significant sequence dissimilarity with representative rodent-, shrew-, mole- and bat-borne hantaviruses, ranging from 30.2–80.5% and 40.2–82.1% at the nucleotide (nt) and amino acid (aa) levels, respectively (Table 3). The novel hantavirus, which was named ASTV 3877 after the location where the Iberian mole was trapped, showed low sequence similarity with two recently described mole-borne hantaviruses: namely, ACDV Academ-Ta450 (S, 54.4% nt/52.2% aa; M, 60.2% nt/52.1% aa; L, 69.1% nt/71.3% aa) and LDRV MNHN-ZM-2017-2257 (S, 50.1% nt/50.8% aa) (Table 3 and Supplemental Table S1).

The full-length 1979-nucleotide S-segment of ASTV 3877 encoded an N protein of 429 amino acids in length. As was in other mole-borne hantaviruses, an additional open reading frame for a nonstructural NSs protein was not present. Our analysis of ASTV also included 1971 and 1369 nt of the M and L segments, respectively, or approximately 50% and 20% of the M and L segments of ASTV.

Of the seven hantavirus samples analyzed by next-generation sequencing, only one (ASTV 3877) had sufficient viral reads to produce viable contigs: 22 contigs from ASTV 3877 exhibited sequence similarity to known hantaviruses. These contigs were translated and aligned to a reference hantavirus (NVAV) to determine which segment they were from and where on the segment they corresponded to. Sequences of ASTV 3877 from next-generation sequences corresponded to that derived from Sanger sequencing.

Compared with other hantaviruses, sequence similarity of a 346-nt region of the L segment amplified from two crowned shrews (SWSV 4050 and SWSV 4056) was 80–85% and 96% at the nucleotide and amino acid levels, respectively, with SWSV.

### 3.3. Phylogenetic Analysis

**Figure 2:**
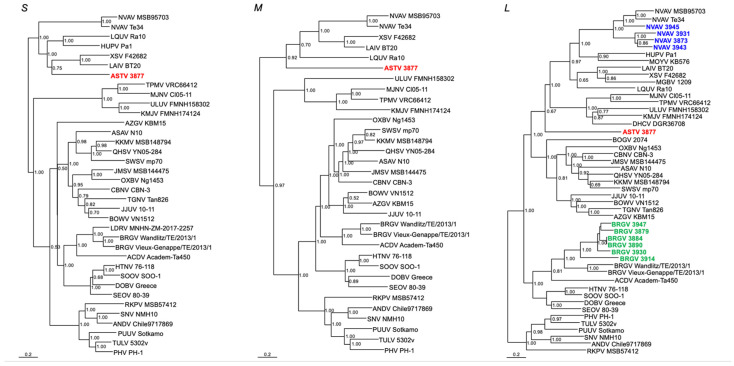
Phylogenetic trees, based on sequences of the S-, M-, and L-genomic segments, respectively, generated by the Bayesian Markov chain Monte Carlo estimation method, under the GTR + I + Γ model of evolution. Hantavirus 3879, 3884, 3890, 3914, 3930 and 3947 (shown in green) from Iberian moles clustered with Bruges virus (BRGV), while 3873, 3931, 3943 and 3945 (shown in blue) clustered with Nova virus (NVAV). Accordingly, these have been named BRGV and NVAV, respectively. By contrast, 3877 (shown in red) was distinct from all other hantaviruses described to date and was named Asturias virus (ASTV). Sequence lengths of the S, M and L segments for ASTV and the L segment of BRGV and NVAV from Iberian moles are shown in Table 2. Also shown are BRGV Vieux-Genappe/TE/2013/1 (S: KX551960; M: KX551961; L: KX551962) and BRGV Wandlitz/TE/2013/1 (S: MF683844; M: MF683845; L: MF683846) from European moles in Belgium and Germany, respectively; ACDV Academ-Ta450 (S: MK340905; M: OL871119; L: MH784614) from a Siberian mole in Russia; and LDRV MNHN-ZM-2017-2257 (S: ON944104) from an Aquitanian mole in France. Other mole-borne hantaviruses include Asama virus (ASAV) N10 (S: EU929072; M: EU929075; L: EU929078) from *Urotrichus talpoides*; Oxbow virus (OXBV) Ng1453 (S: FJ5339166; M: FJ539167; L: FJ593497) from *Neurotrichus gibbsii*; Rockport virus (RKPV) MSB57412 (S: HM015223; M: HM015222; L: HM015221) from *Scalopus aquaticus*; Dahonggou Creek virus (DHCV) DGR36708 (L: HQ616595) from *Scaptonyx fusicaudus*; and Nova virus (NVAV) Te34 (S: KR072621; M: KR072622; L: KR072623) and MSB95703 (S: FJ539168; M: HQ840957; L: FJ593498) from *Talpa europaea*. Bat-borne hantaviruses include Láibīn virus (LAIV) BT20 (S: KM102247; M: KM102248; L: KM102249); Xuân Sơn virus (XSV) F42682 (S: KF704709; M: KJ000538; L: KF704714); Magboi virus (MGBV) 1209 (L: JN037851); Mouyassué virus (MOYV) KB576 (L: JQ287716); Huángpí virus (HUPV) Pa-1 (S: JX473273; L: JX465369); Lóngquán virus (LQUV) Ra10 (S: JX465413; M: JX465396; L: JX465379). Shrew-borne hantaviruses include Cao Bằng virus (CBNV) CBN-3 (S: EF543524; M: EF543526; L: EF543525) from *Anourosorex squamipes*; Jemez Springs virus (JMSV) MSB144475 (S: FJ593499; M: FJ593500; L: FJ593501) from *Sorex monticolus*; Seewis virus (SWSV) mp70 (S: EF636024; M: EF636025; L: EF636026) from *Sorex araneus*; Kenkeme virus (KKMV) MSB148794 (S: GQ306148; M: GQ306149; L: GQ306150) from *Sorex roboratus*; and Qian Hu Shan virus (QHSV) YN05-284 (S: GU566023; M: GU566022; L: GU566021) from *Sorex cylindricauda*; as well as Thottapalayam virus (TPMV) VRC66412 (S: AY526097; M: NC_010708; L: EU001330) from *Suncus murinus*; Imjin virus (MJNV) Cl05-11 (S: EF641804; M: EF641798; L: EF641806) from *Crocidura lasiura*; Azagny virus (AZGV) KBM15 (S: JF276226; M: JF276227; L: JF276228) from *Crocidura obscurior*; Tanganya virus (TGNV) Tan826 (S: EF050455; L: EF050454) from *Crocidura theresea*; Bowé virus (BOWV) VN1512 (S: KC631782; M: KC631783; L: KC631784) from *Crocidura douceti*; Jeju virus (JJUV) SH42 (S: HQ663933; M: HQ663934; L: HQ663935) from *Crocidura shantungensis*; Uluguru virus (ULUV) FMNH158302 (S: JX193695; M: JX193696; L: JX193697) from *Myosorex geata*; and Kilimanjaro virus (KMJV) FMNH174124 (S: JX193698; M: JX193699; L: JX193700) from *Myosorex zinki*. Rodent-borne orthohantaviruses include Sin Nombre virus (SNV) NMH10 (S: NC_005216; M: NC_005215; L: NC_005217); Andes virus (ANDV) Chile9717869 (S: AF291702; M: AF291703; L: AF291704); Prospect Hill virus (PHV) PH-1 (S: Z49098; M: X55129; L: EF646763); Tula virus (TULV) M5302v (S: NC_005227; M: NC_005228; L: NC_005226); Puumala virus (PUUV) Sotkamo (S: NC_005224; M: NC_005223; L: NC_005225); Dobrava virus (DOBV) Greece (S: NC_005233; M: NC_005234; L: NC_005235); Hantaan virus (HTNV) 76-118 (S: NC_005218; M: NC_005219; L: NC_005222); Soochong virus (SOOV) SOO-1 (S: AY675349; M: AY675353; L: DQ056292); and Seoul virus (SEOV) 80-39 (S: NC_005236; M: NC_005237; L: NC_005238). The numbers at selected nodes are Bayesian posterior probabilities (>0.70) based on 150,000 trees. Two replicate Markov chain Monte Carlo runs, consisting of six chains of 10 million generations, each sampled every 100 generations with a burn-in of 25,000 (25%). The scale bars indicate nucleotide substitutions per site.

Phylogenetic analyses based on the full-length S-segment and partial M- and L-segment sequences, using maximum-likelihood and Bayesian methods, indicated that ASTV 3877 represented a distinct hantavirus (Figure 2). 

Analysis of the partial L-segment sequences showed the simultaneous circulation of three distinct hantavirus lineages, which did not segregate according to geography. That is, Iberian moles collected at the same sites showed NVAV mobatvirus (3873, 3931, 3943, 3945) and BRGV orthohantavirus (3879, 3884, 3890, 3914, 3930, 3947) (Figure 2). Posterior node probabilities between BRGV strains from Belgium and Spain and between NVAV strains from Poland and Spain were 1.00 and 1.00, respectively (Figure 2). In addition, phylogenetic analysis of SWSV 4050 and SWSV 4056 from crowned shrews shared a common ancestry with prototype SWSV mp70 and other SWSV strains harbored by soricine shrews (Supplemental Figure S1).

**Figure 3:**
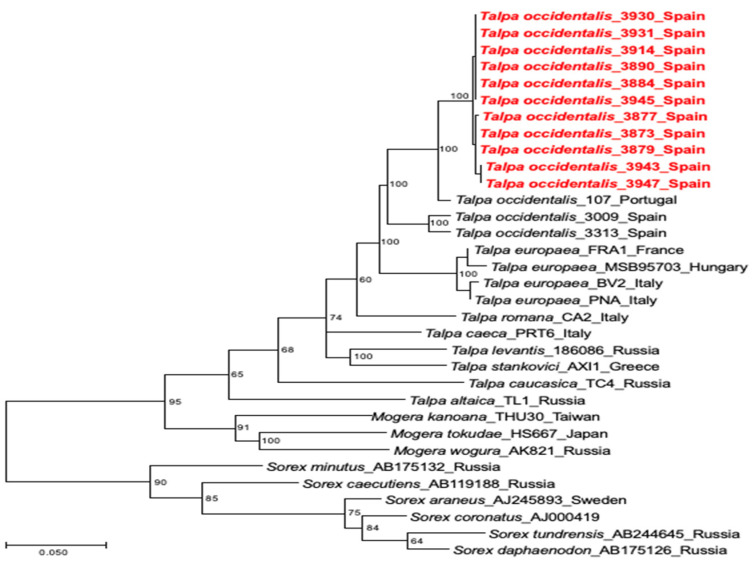
Phylogenetic tree based on full-length sequences of the cytochrome *b* mtDNA using the maximum-likelihood method. The percentage of trees in which the associated taxa clustered together is shown at the respective node. Initial trees for the heuristic search were obtained automatically by applying neighbor-joining and BioNJ algorithms to a matrix of pairwise distances estimated using the maximum composite likelihood (MCL) approach and then selecting the topology with superior log likelihood value. The tree is drawn to scale, with branch lengths measured in the number of substitutions per site. *Talpa occidentalis* from this study are shown in red lettering.

Phylogenetic analysis of the cytochrome *b* mtDNA sequences from the 11 hantavirus RNA-positive moles confirmed the host identity as *Talpa occidentalis* (GenBank accession numbers OQ915046–OQ915056) (Figure 3). 

## 4. Discussion

A genetically distinct hantavirus, designated ASTV, was detected in an Iberian mole captured in northwestern Spain. Iberian moles from Asturias were also shown to harbor BRGV and NVAV. Previously, European moles, the reservoir host of NVAV, was found to harbor BRGV in Belgium, Germany and the United Kingdom [23]. Thus, the co-circulation of ASTV, BRGV and NVAV in Iberian moles was not totally unexpected. With the recent discovery of ACDV [24] and LDRV [25] in the Siberian mole and Aquitania mole, respectively, the number of mole-borne hantaviruses is now nine: four in Europe (ASTV, BRGV, LDRV, NVAV), three in Asia (ACDV, ASAV, DHCV), and two in North America (OXBV, RKPV). Presumably, other hantaviruses are hosted by other mole species or the same mole species. In this regard, the eastern mole in the United States harbors a hantavirus that is distinct from RKPV (H.J. Kang and R. Yanagihara, unpublished observations). 

The European mole, which harbors NVAV and BRGV, has an exceptionally vast geographic range across Europe and western Asia, extending northward to the United Kingdom and southern Scandinavia (Sweden and Finland), southward to northern Greece, and eastward to Poland, Ukraine and western Siberia. By contrast, the Iberian mole has a very limited geographic distribution, and is confined to only Spain and Portugal. Although the European mole is the sole mole species throughout most of its range, there is geographic overlap with the Aquitania mole. Although virus isolation is the gold standard to definitively demonstrate that virus gene amplification findings do not merely represent “spillover” events, the absence of the European mole in Asturias, Spain, would argue strongly against “spillover” and instead would support true infection and co-circulation of NVAV, BRGV and ASTV in the Iberian mole. Curiously, NVAV and BRGV were found more commonly than ASTV among Iberian moles in this study. Only 1 of the 11 hantavirus-infected Iberian moles had ASTV. Nevertheless, a limitation of our study was the failure to include European moles captured west of Asturias and/or elsewhere in northern Spain. 

The detection of SWSV in crowned shrews from Fresnu and Villaviciosa represents the first time SWSV has been reported in this soricine shrew species. SWSV was originally detected in the Eurasian common shrew [20,38,39,40] and subsequently in the Laxmann’s shrew (*Sorex caecutiens*) [41], large-toothed Siberian shrew (*Sorex daphaenodon*) [40], Eurasian pygmy shrew (*Sorex minutus*) [20], tundra shrew (*Sorex tundrensis*) [40], and Mediterranean water shrew (*Neomys anomalus*) [20]. Thus, as previously noted, a given hantavirus species may be hosted by multiple species of closely related rodents, shrews and moles [12,42,43]. Conversely, a given small-mammal host species is capable of serving as the reservoir of more than one hantavirus species. For example, the Eurasian common shrew hosts SWSV and Altai virus (ALTV) [44,45], and the striped field mouse (*Apodemus agrarius*) hosts Hantaan orthohantavirus [1,46] and Dobrava–Belgrade orthohantavirus [47,48,49].

In addition, reports of sympatric virus species in sympatric hosts [50,51] and reassortment [52,53,54] emphasize the complex evolutionary history of hantaviruses. 

Interestingly, while analyzing cytochrome b mtDNA sequences of 85 European moles from 46 localities across nearly all of its geographic range, Feuda and colleagues found three differentiated mtDNA lineages, of which two were restricted to Spain and Italy and a third that was widespread across Europe [55]. Phylogenetic inferences and molecular clock analysis suggested that the European moles from Spain represented a highly divergent and ancient lineage.

Recently, in a reanalysis of cytochrome *b* mtDNA sequences from European moles from west and south of the Loire River in France and from northern Spain, a new mole species, tentatively named the Aquitanian mole (*Talpa aquitania*), was reported [56]. The Aquitanian mole is the reservoir host of a newly described hantavirus (LDRV) [25]. Unfortunately, our study did not include Aquitanian moles, as evidenced by mtDNA sequence analysis, which confirmed that all ASTV-, NVAV- and BRGV-infected moles were, in fact, Iberian moles. Nevertheless, future studies are warranted to analyze tissues from European moles and Aquitanian moles in northern Spain for LDRV and other hantaviruses.

A recently published proposal would require full coding sequences for classification of hantaviruses [57]. If approved by the International Committee on Taxonomy of Viruses, this will present obvious challenges for multiple well-known “classical” rodent-borne orthohantaviruses that have yet to be fully sequenced, as well as for many of the newfound hantaviruses detected in shrews, moles and bats. To date, non-rodent-borne hantavirus isolates exist only for Thottapalayam thottimvirus, Imjin thottimvirus and NVAV. Of the more than 30 recently described shrew-, mole- and bat-borne hantaviruses, none have been isolated in cell culture and most exist only as partial sequences. 

## 5. Conclusions

The view that each hantavirus species is harbored by a single small-mammal host species now appears overly simplistic and invalid [58,59]. Host sharing, host-switching and spill-over events have occurred frequently in the evolutionary history of hantaviruses such that certain hantavirus species are carried by multiple sympatric small mammal hosts, and conversely, certain reservoir species may host more than one hantavirus species. Like the European mole, which hosts NVAV and BRGV, Iberian moles appear to harbor more than one hantavirus species. The definitive identification of hantaviruses in the Iberian mole must await whole-genome sequence analysis and virus isolation. Concurrently, sympatric soricine and crocidurine shrews need to be investigated to determine if they may represent the sources of hantavirus diversity in Iberian moles. Overall, this report supports the growing understanding of hantavirus and host ecology as an evolutionarily complex history that merits continued research into the patterns and mechanisms involved.

## Figures and Tables

**Table 1:** RT-PCR detection of hantaviruses, using L-segment primers, in small mammals in Asturias, Spain.

Collection Site	Year	*Talpa * *occidentalis*	*Crocidura russula*	*Sorex * *coronatus*	*Microtus * *lusitanicus*	*Arvicola scherman*
Coceña	2011	3/11	0/2		0/2	
	2012				0/1	
Fresnadiellu	2012	2/9				
	2013					0/6
Fresnu	2012	0/2		1/5	0/3	0/1
Oles	2011	1/15			0/2	
	2012	1/3				
	2013	1/2		0/1		0/4
	2014			0/1		
Priesca	2011	3/13			0/1	
	2012	0/2			0/2	
	2013	0/1				0/1
	2014					0/3
VEV (Villaviciosa)	2011		0/2	1/4		

**Table 2:** Hantaviruses detected in Iberian moles in Asturias, Spain.

Virus	Collection Site	Nucleotides and GenBank Accession Numbers		
		S Segment	M Segment	L Segment
ASTV 3877	Coceña	1979 bp KY040518	1971 bp KY040519	1369 bp KY040520
BRGV 3879	Coceña			1327 bp KY040512
BRGV 3884	Fresnadiellu			1327 bp KY040513
BRGV 3890	Fresnadiellu			553 bp KY040514
BRGV 3914	Oles			553 bp KY040515
BRGV 3930	Oles			553 bp KY040516
BRGV 3947	Priesca			1327 bp KY040517
NVAV 3873	Coceña			693 bp KY040508
NVAV 3931	Oles			352 bp KY040509
NVAV 3943	Priesca			352 bp KY040510
NVAV 3945	Priesca			977 bp KY040511

**Table 3:** Nucleotide and amino acid sequence similarities (%) between ASTV 3877 and other representative mole-, shrew-, bat- and rodent-borne hantaviruses.

		S Segment		M Segment		L Segment	
Host	Hantavirus	1979 nt	429 aa	1971 nt	657 aa	1369 nt	456 aa
Mole	ACDV Academ-Ta450	54.4	52.2	60.2	52.1	69.1	71.3
	ASAV N10	49.5	48.7	59.5	49.0	69.2	71.3
	**BRGV 3879**	-	-	-	-	67.7	69.5
	**BRGV 3884**	-	-	-	-	67.2	69.9
	**BRGV 3890**	-	-	-	-	69.8	73.9
	**BRGV 3914**	-	-	-	-	80.5	82.1
	**BRGV 3930**	-	-	-	-	70.2	73.9
	**BRGV 3947**	-	-	-	-	69.0	70.8
	BRGV Vieux-Genappe/TE/2013/1	52.0	51.3	59.3	49.8	67.8	69.5
	BRGV Wandlitz/TE/2013/1	52.6	51.0	59.6	49.8	66.3	68.9
	DHCV DGR36708	-	-	-	-	66.0	66.5
	LDRV MNHN-ZM-2017-2257	50.1	50.8	-	-	-	-
	**NVAV 3873**	-	-	-	-	39.4	44.2
	**NVAV 3931**	-	-	-	-	69.9	68.4
	**NVAV 3943**	-	-	-	-	70.7	69.2
	**NVAV 3945**	-	-	-	-	64.8	66.2
	NVAV MSB95703	61.3	56.3	60.6	53.3	67.1	69.3
	NVAV Te34	62.1	56.1	62.5	55.3	67.5	68.9
	OXBV Ng1453	59.6	49.1	59.3	47.6	69.2	68.9
	RKPV MSB57412	60.4	53.0	58.9	49.0	67.3	69.1
Shrew	MJNV Cl05-11	51.4	45.7	59.8	48.4	66.1	68.6
	TPMV VRC66412	47.9	48.0	59.2	49.2	67.2	69.3
	ULUV FMNH158302	61.8	50.9	57.4	37.7	66.8	67.1
	KMJV FMNH174124	59.6	51.9	57.7	40.2	67.8	69.3
	BOGV 2074	-	-	-	-	68.5	71.9
	JMSV MSB144475	49.6	47.6	58.6	50.4	67.9	69.3
	KKMV MSB148794	50.7	50.8	53.1	46.1	67.6	71.3
	SWSV mp70	51.0	50.3	61.4	57.8	68.0	70.0
	**SWSV 4050**	-	-	-	-	67.1	73.0
	**SWSV 4056**	-	-	-	-	67.9	73.0
	QHSV YN05-284	49.3	50.6	60.5	49.2	60.0	62.4
	CBNV CBN-3	50.6	50.2	61.1	50.5	68.2	70.6
	JJUV 10-11	52.5	48.4	60.9	49.0	66.5	67.5
	TGNV Tan826	30.4	46.3	-	-	59.6	58.4
	AZGV KBM15	30.2	48.3	55.7	45.9	67.4	67.5
	BOWV VN1512	60.1	48.7	59.4	47.5	67.9	68.2
Bat	XSV F42682	62.6	56.2	59.8	53.8	47.2	50.3
	LAIV BT20	63.5	58.1	62.2	56.0	68.3	72.6
	LQUV Ra10	63.3	54.4	60.3	47.6	69.1	71.3
	HUPV Pa1	62.5	56.1	-	-	66.5	74.6
	MOYV KB576	-	-	-	-	67.6	71.1
	MGBV 1209	-	-	-	-	62.6	61.3
Rodent	HTNV 76-118	53.5	53.1	57.7	51.8	67.0	67.5
	SEOV 80-39	49.5	51.5	59.7	51.6	67.1	70.6
	SOOV SOO-1	54.2	53.4	59.3	51.0	67.6	68.2
	DOBV Greece	50.9	53.1	60.5	51.9	68.3	70.8
	ANDV Chile9717869	59.1	53.3	60.1	49.9	66.0	66.0
	SNV NMH10	58.0	53.5	59.5	49.6	66.4	67.8
	PUUV Sotkamo	61.5	52.2	59.8	50.8	67.6	68.0
	TULV 5302v	58.1	53.1	61.0	49.8	67.7	66.2
	PHV PH-1	61.9	51.5	59.5	49.5	65.4	66.7

Abbreviations: ACDV, Academ virus; ANDV, Andes virus; ASAV, Asama virus; AZGV, Azagny virus; BOGV, Boginia virus; BOWV, Bowé virus; BRGV, Bruges virus; CBNV, Cao Bằng virus; DOBV, Dobrava virus; DHCV, Dahonggou Creek virus; HTNV, Hantaan virus; HUPV, Huángpí virus; JMSV, Jemez Spring virus; JJUV, Jeju virus; KKMV, Kenkeme virus; KMJV, Kilimanjaro virus; LAIV, Láibīn virus; LDRV, Landiras virus; LQUV, Lóngquán virus; MGBV, Magboi virus; MJNV, Imjin virus; MOYV, Mouyassué virus; NVAV, Nova virus; OXBV, Oxbow virus; PHV, Prospect Hill virus; PUUV, Puumala virus; QHSV, Qian Hu Shan virus; RKPV, Rockport virus; SEOV, Seoul virus; SNV, Sin Nombre virus; SOOV, Soochong virus; SWSV, Seewis virus; TGNV, Tanganya virus; TPMV, Thottapalayam virus; TULV, Tula virus; ULUV, Uluguru virus; XSV, Xuân Sơn virus. nt, Nucleotides; aa, amino acids. Newfound hantaviruses are shown in bold text.

## Data Availability

GenBank accession numbers for phylogenetic analyses are available in Table 2 and in the legend of Figure 2. Other presented data are available on request from the corresponding author.

## Supplementary Materials

The following supporting information can be downloaded at: https://www.mdpi.com/article/10.3390/v15061313/s1, Table S1: Pairwise comparisons of nucleotide and amino acid sequence similarities of the S, M and L segments of Asturias virus (ASTV) and other mole-, shrew-, bat- and rodent-borne hantaviruses; Figure S1: Phylogenetic tree of Seewis virus (SWSV) detected in the crowned shrew (*Sorex coronatus*).
