# Supplementary material for: Multiple Lineages of Hantaviruses Harbored by the Iberian Mole (Talpa occidentalis) in Spain
Source: Viruses. 2023 Jun 2;15(6):1313. doi: 10.3390/v15061313 (PMC10302183; doi:10.3390/v15061313)
Supplement: Supplementary file 1 [file viruses-15-01313-s001.zip › ASTV Supplemental Figure S1 R1.pdf]

ML tree based on the L segment (346 bp)

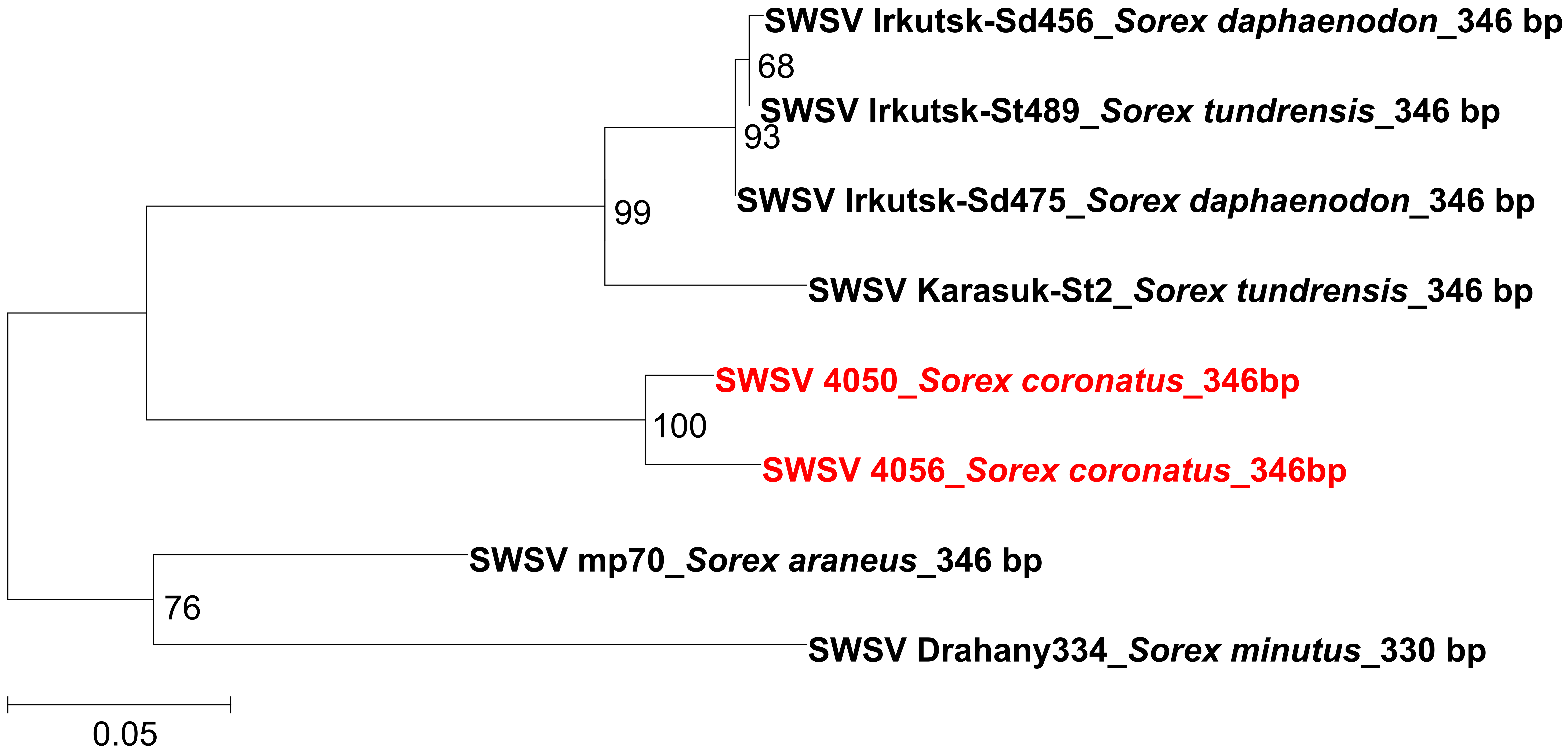

Supplemental Figure S1. Phlyogenetic tree based on partial L-segment sequences of SWSV 4050 and SWSV 4056 and other SWSV strains harbored by other soricine shrew species, using the maximum-likelihood method. The percentage of trees in which the associated taxa clustered together is shown next to the branches. Initial trees for the heuristic search were obtained automatically by applying Neighbor-Join and BioNJ algorithms to a matrix of pairwise distances estimated using the Maximum Composite Likelihood (MCL) approach, and then selecting the topology with supe-rior log likelihood value. The tree is drawn to scale, with branch lengths measured in the number of substitutions per site.
