# Supplementary material for: Multiple Lineages of Hantaviruses Harbored by the Iberian Mole (Talpa occidentalis) in Spain
Source: Viruses. 2023 Jun 2;15(6):1313. doi: 10.3390/v15061313 (PMC10302183; doi:10.3390/v15061313)
Supplement: Supplementary file 1 [file viruses-15-01313-s001.zip › ASTV Supplemental Table S1 R1.pdf]

S seg pairwise table

MegAlign - [Pair Distances of 230425_ASTV_Snt_final.meg ClustalW (Slow/Accurate, IUB)]

File Edit Align View Options Net Search Help

Percent Identity

Divergence

	1	2	3	4	5	6	7	8	9	10	11	12	13	14	15	16	17	18	19	20	21	22	23	24	25	26	27	28	29	30	31	32	33	34	35	36		
1		62.1	61.3	62.6	52.6	52.0	50.1	54.4	63.5	63.3	62.5	51.4	47.9	61.8	59.6	49.5	59.6	49.6	51.0	49.3	50.7	50.6	52.5	29.0	37.6	60.1	60.4	53.5	54.2	49.5	50.9	59.1	58.0	61.5	58.1	61.9	1	3877_entireSorf_1287bp
2	56.8		86.4	59.3	51.6	53.0	51.2	54.2	57.9	54.9	61.6	50.2	49.1	62.0	50.8	50.9	51.5	51.4	51.6	53.0	58.4	57.2	50.9	64.0	39.4	59.1	57.9	50.4	56.6	57.7	57.5	53.4	51.3	58.6	51.6	50.1	2	NVAV Te34_entireS_1825bp
3	57.5	15.4		60.6	51.9	52.7	51.4	52.7	53.5	55.3	62.7	50.5	49.5	61.1	49.2	51.1	58.6	57.6	51.3	52.6	49.6	58.3	51.5	61.8	39.3	48.9	51.4	55.6	56.9	55.7	56.7	59.2	57.2	59.3	56.9	52.0	3	NVAV_MSB95703_S_entire
4	59.4	63.1	58.4		58.2	59.5	59.9	62.1	72.5	64.1	62.4	48.9	47.5	56.4	50.0	51.8	50.0	52.3	52.0	49.3	58.1	58.8	57.1	62.9	41.7	62.2	52.6	62.2	62.1	61.2	54.2	51.6	51.5	58.2	59.9	58.2	4	XSV 2589_S entire_1752bp
5	65.6	68.3	67.7	65.5		82.2	73.7	69.3	52.0	51.9	60.3	50.5	48.1	49.3	50.1	64.2	65.7	65.8	55.7	64.2	64.4	66.4	65.6	63.1	40.0	65.1	65.1	65.5	65.3	65.3	64.3	63.6	64.1	64.3	54.0	64.1	5	BRGV_DE_MF683844_Sorf
6	65.2	66.1	65.0	60.5	20.6		76.6	70.7	51.5	53.0	62.4	48.4	50.2	51.4	49.5	64.0	64.7	63.9	64.3	55.5	63.6	66.3	65.7	63.3	41.3	65.3	64.4	65.7	66.9	65.0	65.1	64.6	63.5	54.3	54.3	54.3	6	BRGV_BE_KX551961_Sorf
7	67.4	68.8	68.0	61.2	32.8	29.0		69.2	61.8	54.4	62.0	50.9	48.8	47.5	50.4	65.6	65.5	64.8	64.4	63.3	63.7	67.1	65.2	62.4	63.5	66.1	65.0	65.7	67.0	65.0	65.6	54.7	61.5	62.2	62.2	63.6	7	LDRV_FR_Sorf
8	61.8	62.4	63.5	59.1	42.4	38.9	41.5		62.0	56.7	60.1	49.8	48.4	49.6	60.4	64.7	67.9	67.4	65.3	63.9	65.1	66.1	66.3	68.6	64.1	65.7	65.5	67.7	68.3	65.7	66.6	65.0	64.8	63.6	63.3	64.3	8	ACDV_Ta450_Sorf
9	58.3	62.2	60.7	35.7	64.1	60.9	61.3	59.9		67.3	63.2	47.2	49.5	58.2	58.4	52.0	51.1	51.1	51.9	49.0	50.7	60.6	62.5	62.7	41.9	61.0	57.4	50.3	61.3	50.0	55.0	55.8	51.4	63.3	56.0	59.3	9	LBV S_ORF
10	59.5	66.3	64.4	56.9	66.4	64.8	60.8	62.1	51.8		63.2	48.8	50.7	48.2	57.1	51.1	51.0	58.1	51.1	51.0	50.4	52.4	61.6	44.8	65.7	59.5	56.2	50.6	60.8	52.5	49.5	54.1	51.3	51.8	51.1	51.2	10	LQUVRa10 S_ORF
11	58.4	59.0	56.9	54.1	61.6	59.2	60.7	57.1	51.9	50.2		59.0	58.1	48.3	56.0	59.4	61.7	64.1	61.4	61.5	61.0	62.4	62.4	60.9	41.1	61.6	63.8	59.7	63.8	61.9	62.5	59.8	60.9	61.9	63.1	61.5	11	Huangpi_Pa_1_S
12	68.2	74.5	75.6	69.9	73.8	73.2	74.2	74.0	74.6	76.2	65.3		65.5	56.2	66.9	49.9	48.8	49.7	48.6	50.5	47.8	51.2	60.5	58.6	38.3	48.7	48.7	50.1	50.1	51.0	47.8	50.6	51.9	49.6	48.3	50.8	12	MJNV S_ORF
13	72.2	74.7	75.9	73.6	78.8	75.7	73.3	73.7	70.1	75.7	68.2	43.5		53.3	67.6	49.3	47.6	49.7	47.5	46.8	48.6	49.8	47.9	25.8	35.7	48.7	50.2	54.3	54.3	48.4	49.7	52.5	48.9	50.8	50.1	53.0	13	TPMV S_ORF
14	63.1	68.4	71.1	64.8	76.1	70.8	74.8	70.0	67.1	69.0	76.2	50.0	55.5		71.2	50.8	46.3	49.8	49.2	50.4	50.2	50.7	48.4	26.7	37.2	47.2	49.5	48.8	48.3	50.9	47.7	48.0	50.5	51.5	48.3	51.9	14	ULU FMNH158302_ORF S
15	66.9	71.3	70.0	68.7	75.3	75.2	72.7	70.6	66.9	71.2	69.2	47.6	45.3	37.5		46.1	50.3	50.4	47.4	49.3	48.5	51.1	50.4	27.4	45.4	50.4	49.8	49.7	52.8	50.9	48.1	51.2	51.8	53.2	49.6	51.4	15	KMJ FMNH174124_entire ORF S
16	72.0	67.0	66.3	66.5	55.5	55.1	53.5	54.1	67.0	65.0	61.0	68.6	69.9	71.9	72.8		67.9	69.2	63.5	69.7	68.3	69.2	67.1	68.6	66.9	68.8	54.9	62.5	66.4	65.7	64.9	64.7	64.3	63.4	53.7	63.6	16	ASAV S_ORF
17	63.5	64.0	64.1	66.3	53.7	54.7	53.4	45.4	64.3	61.9	58.0	76.4	76.5	74.0	72.8	44.6		74.9	64.4	65.3	67.6	69.7	66.5	65.2	66.3	67.8	65.1	64.2	65.1	64.4	64.9	64.8	63.2	65.7	63.6	62.0	17	OXBV S_ORF
18	65.9	64.9	63.4	62.4	52.5	55.3	53.1	47.9	63.0	60.2	53.9	74.6	70.3	73.1	70.0	43.3	30.9		69.1	70.6	63.1	71.7	68.8	66.3	66.5	69.6	64.5	67.3	66.2	65.9	65.7	64.6	62.6	63.9	60.5	65.2	18	JMSV S_ORF
19	65.4	62.9	65.0	65.7	54.4	54.7	57.7	50.1	65.0	70.2	55.9	72.4	80.1	70.3	72.9	46.6	50.9	42.8		72.7	74.7	65.5	62.3	69.2	43.3	67.4	54.3	54.9	54.6	53.1	62.5	56.3	55.5	57.0	57.5	55.4	19	SWSV S_ORF
20	66.0	63.9	63.0	62.7	55.8	54.5	56.1	53.9	68.6	64.7	55.7	69.4	75.2	70.6	73.0	41.0	50.2	41.5	34.7		75.4	67.3	60.8	65.4	65.7	67.3	62.8	65.0	63.7	62.5	66.2	56.6	61.8	53.7	53.1	55.3	20	QDLV S_ORF
21	64.3	64.9	68.3	69.4	53.4	55.2	56.6	52.3	68.0	67.0	55.7	72.2	74.7	74.0	73.8	46.0	47.7	42.9	30.8	30.0		66.1	65.3	67.9	46.5	65.9	64.6	63.3	64.6	54.1	63.4	54.8	60.6	55.1	61.2	56.6	21	KKMV S_ORF
22	63.9	65.0	64.1	61.4	51.5	52.4	48.6	46.5	62.0	62.9	57.7	67.9	66.0	69.3	67.6	42.9	41.1	36.8	49.0	47.4	50.4		69.6	65.8	68.1	69.2	64.3	65.1	65.4	64.0	65.0	63.8	53.9	64.3	63.0	62.6	22	CBNV S_ORF
23	68.3	66.0	64.5	62.6	53.8	52.9	50.9	49.1	61.0	60.8	54.7	70.6	70.3	71.3	72.0	47.5	48.1	42.4	50.8	52.8	48.3	40.2		67.0	69.1	71.0	63.1	65.1	66.0	65.2	63.7	61.1	62.8	64.9	64.2	54.4	23	JJUV S_ORF
24	68.5	61.8	68.0	62.5	63.7	60.1	66.5	46.9	59.9	65.5	66.3	71.0	82.6	75.2	76.7	45.4	49.2	48.7	44.8	53.0	48.1	50.2	46.1		63.1	71.5	63.8	34.4	65.2	37.3	62.2	61.5	33.3	67.6	29.6	65.8	24	TGNV S_ORF
25	62.9	65.3	62.0	59.3	61.0	65.4	57.9	57.5	59.2	45.7	63.6	75.2	70.4	62.1	67.6	53.3	51.8	50.1	59.5	49.9	51.6	47.5	44.0	51.3		65.4	67.6	42.6	41.9	55.4	55.2	44.4	42.8	67.0	43.5	67.6	25	AZGV S_ORF
26	63.3	63.5	67.1	61.6	52.5	52.6	49.7	48.8	61.3	64.0	55.6	71.0	72.8	67.5	69.3	43.2	45.8	41.3	48.1	44.6	43.1	41.8	37.5	38.8	47.2		64.3	66.4	64.0	63.9	65.0	65.0	61.4	63.3	63.2	63.3	26	BOWV S_ORF
27	64.5	64.6	64.8	64.0	53.9	57.0	58.7	51.6	58.3	58.6	55.8	72.8	71.3	71.2	68.9	58.2	56.4	55.1	56.6	57.2	55.6	56.3	59.1	65.0	49.5	56.2		64.5	63.6	65.2	65.8	71.3	69.6	69.1	70.6	69.9	27	RKPV S_ORF
28	62.4	67.1	67.6	59.5	51.4	50.5	50.5	46.3	63.1	66.7	57.8	68.3	70.3	73.1	69.4	56.9	53.7	46.0	55.2	50.2	54.8	50.9	50.9	60.5	57.9	48.5	53.1		82.9	74.8	74.5	66.5	64.3	63.9	64.3	66.6	28	HTNV S_ORF
29	65.8	66.8	66.0	61.5	52.2	48.5	49.5	45.5	62.3	63.7	51.7	66.5	70.5	72.6	68.6	50.4	53.4	51.0	55.1	54.7	54.3	50.6	51.2	60.7	60.2	53.7	54.1	19.8		74.2	73.7	65.5	64.7	64.9	66.0	65.2	29	SOOV S_ORF
30	66.5	65.0	69.5	62.9	52.7	51.8	54.2	51.0	65.4	70.6	55.0	67.2	71.5	73.0	67.0	52.3	52.5	49.2	56.7	54.2	55.4	54.8	52.5	57.9	53.8	53.1	51.9	30.5	31.9		74.4	66.4	65.4	63.6	65.3	65.3	30	SEOV S_ORF
31	64.7	65.5	67.0	62.9	52.6	52.5	50.7	49.5	62.0	65.9	53.6	70.4	70.1	73.7	69.2	53.0	52.9	50.6	56.0	53.1	57.3	51.1	56.3	64.9	53.3	54.7	52.0	32.4	33.7	31.9		65.3	65.9	64.0	64.3	65.3	31	DOBV S_ORF
32	65.8	66.8	68.1	61.3	56.3	55.5	58.2	54.7	63.4	65.6	56.3	68.8	64.8	70.9	65.0	57.1	56.0	51.9	56.1	54.3	59.9	52.1	59.7	67.9	52.6	56.0	37.5	50.4	51.5	50.3	53.8		77.0	70.9	70.7	73.0	32	ANDV S_ORF
33	66.1	66.3	66.5	63.0	58.9	57.4	58.8	56.1	64.4	64.7	57.3	68.5	70.8	66.8	66.7	55.9	60.4	55.8	60.3	57.2	59.0	56.2	59.5	66.1	59.9	58.6	39.6	51.0	51.3	50.2	50.1	28.3		69.6	72.1	71.0	33	SNV S_ORF
34	63.7	63.4	61.5	58.5	55.8	57.4	60.6	52.3	59.8	65.2	57.8	69.3	67.6	65.8	63.9	57.0	55.2	53.4	56.8	56.8	56.9	55.7	54.2	59.6	51.4	54.3	43.7	55.1	54.2	56.9	53.6	39.9	41.7		74.7	73.0	34	PUUV S_ORF
35	63.7	65.8	65.0	56.5	59.9	57.8	59.7	54.4	62.4	63.0	57.1	70.3	69.3	70.5	70.6	56.9	56.3	54.6	55.8	60.1	56.8	57.8	57.1	63.6	54.1	56.8	39.9	50.6	47.7	51.0	53.0	39.8	36.3	33.1		76.2	35	TULV S_ORF
36	64.4	67.4	64.5	58.7	54.7	58.1	55.9	54.1	63.0	65.3	60.8	71.9	68.8	67.9	68.5	56.9	60.3	50.2	59.0	58.4	60.0	57.0	57.7	61.0	49.6	56.2	40.8	51.3	53.6	51.0	51.5	36.8	38.7	34.3	29.4		36	PHV S_ORF
	1	2	3	4	5	6	7	8	9	10	11	12	13	14	15	16	17	18	19	20	21	22	23	24	25	26	27	28	29	30	31	32	33	34	35	36		

M seg pairwise table

MegAlign - [Pair Distances of 230425_ASTV_Mnt_final.meg ClustalW (Slow/Accurate, IUB)]

File Edit Align View Options Net Search Help

Percent Identity

Divergence

	1	2	3	4	5	6	7	8	9	10	11	12	13	14	15	16	17	18	19	20	21	22	23	24	25	26	27	28	29	30	31	32	33		
1		62.5	60.6	59.6	59.3	60.2	59.7	62.2	60.3	59.8	59.2	48.8	49.5	59.5	59.3	58.6	61.4	60.5	53.1	61.1	60.9	55.7	59.4	58.9	57.7	59.7	59.3	60.5	60.1	59.5	59.8	61.0	59.5	1	3877_M_1971bp
2	56.4		82.4	53.5	56.2	54.8	63.2	63.5	44.3	41.2	43.5	55.9	49.7	54.6	54.4	60.0	54.6	61.2	57.3	46.8	52.4	55.6	44.9	54.3	52.8	42.9	50.6	43.4	42.9	43.4	45.6	57.2	59.1	2	NVAV_Te34 M_ORF
3	65.4	20.4		51.7	52.5	51.4	16.0	61.5	57.6	57.2	51.0	53.5	60.6	50.4	50.7	57.7	8.4	55.4	47.0	57.6	53.8	56.3	54.9	55.4	53.7	56.3	51.7	55.8	54.9	56.6	56.6	55.1	55.6	3	NVAV M_ORF
4	62.8	70.2	76.4		81.4	69.6	58.4	53.9	44.9	41.1	41.5	51.4	50.1	64.9	63.1	67.7	67.9	67.1	64.9	65.1	61.3	64.6	62.3	61.3	63.5	63.8	62.3	60.9	56.3	60.4	60.1	60.6	60.1	4	BRGV_DE_MF683845_Morf
5	64.0	70.7	78.5	21.8		68.6	60.2	53.0	52.9	40.7	41.1	48.1	48.1	63.9	62.9	68.7	70.3	66.4	64.8	64.4	61.5	62.3	63.3	60.8	63.2	63.2	63.3	62.1	58.1	60.4	60.0	61.3	60.6	5	BRGV_BE_KX551961_Morf
6	60.6	70.7	75.0	39.6	41.4		61.5	53.3	52.4	39.5	41.7	53.7	45.9	64.2	63.0	68.3	66.3	67.8	66.3	63.9	61.7	61.0	63.2	57.1	62.1	62.6	62.9	62.2	59.7	58.6	58.9	61.9	57.8	6	ACDV_Ta450_Morf
7	58.0	50.4	68.1	63.1	57.9	55.0		70.1	61.5	56.1	55.5	31.7	14.0	60.3	61.1	59.6	6.0	61.4	48.6	57.5	60.5	9.0	58.4	58.1	59.9	59.0	57.3	56.9	56.9	59.6	59.1	58.5	62.3	7	XSV 2589_M663bp
8	55.2	54.4	63.7	70.3	70.5	69.0	36.6		60.0	41.5	41.7	54.4	51.6	55.7	54.2	61.6	63.1	61.4	50.2	48.0	54.2	58.7	52.3	56.9	53.3	45.6	45.9	53.8	55.0	56.2	53.4	57.9	55.8	8	LBV M_ORF
9	62.8	71.0	75.4	80.4	77.2	80.2	55.6	67.2		41.6	41.5	55.3	46.1	53.5	44.8	59.1	52.2	60.8	54.1	55.0	39.9	54.3	52.4	58.0	51.7	40.2	41.3	40.3	52.6	41.2	39.7	41.4	54.7	9	LQUV_Ra10M_ORF
10	64.4	78.5	72.5	78.6	77.2	80.5	71.0	77.0	79.1		68.0	58.6	64.9	42.0	40.7	59.1	65.1	60.8	60.0	43.2	40.8	54.9	41.5	41.6	41.6	41.4	40.7	41.7	43.4	40.6	40.8	40.8	40.5	10	MJNV M_ORF
11	66.3	76.7	74.2	78.1	77.9	76.4	75.6	77.4	82.1	40.8		61.5	65.1	42.1	41.2	60.3	56.2	61.6	57.4	41.6	41.2	56.2	40.5	40.8	40.7	41.0	40.9	41.6	41.5	41.1	41.1	41.2	41.6	11	TPMV M_ORF
12	67.6	68.3	72.3	77.4	73.6	78.4	65.7	68.9	69.6	57.7	57.5		59.2	49.7	48.3	43.8	43.8	45.6	42.3	49.1	53.9	49.6	50.1	52.5	40.8	51.7	54.2	52.2	48.8	49.2	48.9	49.1	48.5	12	ULU FMNH158302_partial M
13	63.7	64.9	61.8	73.3	76.3	77.5	72.1	66.3	73.7	47.8	45.9	60.4		49.1	48.2	44.0	52.2	41.6	38.8	46.8	43.8	54.3	50.9	46.5	46.1	45.2	45.2	49.1	46.2	47.6	50.2	49.3	47.8	13	KMJ FMNH174124_partial M
14	64.2	67.3	77.8	49.9	50.4	50.5	67.7	69.7	75.5	78.5	75.6	77.7	71.5		66.6	73.4	72.7	73.7	75.2	68.9	66.8	62.7	67.9	60.6	63.4	63.4	63.7	63.5	60.8	60.1	59.6	61.4	59.4	14	ASAV M_ORF
15	63.6	69.4	78.9	52.9	53.5	54.8	59.6	68.5	77.0	81.8	78.7	78.3	74.2	44.8		70.7	73.1	69.0	67.9	68.1	65.5	63.8	64.7	59.2	63.4	63.2	63.4	61.6	57.1	59.3	60.2	59.4	57.3	15	OXBV M_ORF
16	64.2	62.8	76.4	43.1	42.9	42.3	59.9	61.1	68.4	65.2	63.0	73.8	64.6	34.8	39.4		72.7	73.3	75.0	74.3	71.2	69.4	70.2	64.5	70.8	69.9	69.9	68.3	62.7	61.7	64.6	64.1	63.9	16	JMSV M_ORF
17	56.0	56.7	45.1	38.8	39.1	41.5	0.0	50.2	68.9	53.4	54.7	74.7	60.1	29.8	33.5	34.1		77.1	15.7	76.7	67.9	10.4	71.5	57.0	66.7	70.3	73.5	67.9	57.4	65.9	60.6	62.2	59.4	17	SWSV M_ORF
18	63.8	60.0	70.5	45.0	47.4	44.4	59.4	62.3	66.2	61.7	63.1	72.7	65.0	33.2	42.4	33.4	27.6		75.0	72.1	69.0	68.6	68.5	63.9	66.9	68.7	68.8	68.6	64.2	64.3	62.1	61.9	60.4	18	QSHV M_ORF
19	68.8	68.0	85.8	48.9	48.6	48.2	64.9	70.8	72.0	65.4	71.0	74.1	77.6	30.6	45.0	31.8	0.0	31.0		71.4	69.1	64.3	70.9	65.1	68.3	67.7	69.1	66.0	56.2	57.4	57.5	56.7	55.8	19	KKMV M_ORF
20	60.4	70.8	69.0	47.9	50.2	49.6	64.3	69.9	74.1	79.9	77.4	78.0	71.1	41.6	44.3	32.8	28.3	35.8	37.3		66.3	69.9	66.7	59.2	63.9	64.3	64.1	63.8	61.6	61.5	59.1	60.9	58.2	20	CBNV M_ORF
21	61.7	70.2	73.4	56.1	55.9	57.5	61.7	72.2	76.2	80.1	82.0	73.2	75.7	47.7	50.8	37.6	42.7	40.9	41.1	47.9		71.3	69.9	56.1	62.8	63.3	63.0	63.2	55.1	58.4	59.1	59.1	59.5	21	JJUV M_ORF
22	73.7	78.6	77.2	49.2	49.8	48.4	86.0	71.8	79.3	70.3	74.7	82.3	74.5	45.9	45.7	40.2	35.6	41.6	38.4	38.0	36.6		73.1	60.4	66.7	67.2	65.6	70.0	60.6	61.4	62.3	62.9	58.1	22	AZGV M_ORF
23	65.8	73.4	69.6	54.7	54.0	54.5	64.2	71.3	77.7	80.6	80.7	75.9	72.4	45.7	50.1	38.3	36.6	42.0	39.0	45.3	39.1	33.7		62.1	62.9	61.2	63.3	62.6	59.6	59.3	60.5	59.2	59.2	23	BOWV M_ORF
24	64.5	66.9	70.7	61.8	61.0	63.8	63.3	66.3	70.6	77.9	77.7	72.6	76.7	61.4	62.7	51.8	54.4	55.1	54.7	61.3	62.1	61.6	61.8		60.9	55.9	60.9	58.8	63.2	64.5	64.3	64.5	62.8	24	RKPV M_ORF
25	64.3	72.1	74.2	53.9	54.8	53.1	58.2	69.0	80.6	80.0	80.6	80.1	76.2	53.4	52.8	38.9	40.2	44.4	42.3	52.4	56.1	44.2	54.9	60.6		72.4	80.5	71.1	60.4	60.4	60.8	61.1	61.3	25	HTNV M_ORF
26	63.4	70.5	69.2	54.6	54.5	54.3	59.0	72.5	78.8	76.6	79.5	77.7	78.1	53.9	55.3	40.7	35.5	42.0	44.1	52.4	56.7	45.8	56.4	62.4	35.5		72.2	71.6	60.9	60.2	60.7	61.3	55.6	26	SEOV M_ORF
27	63.6	69.6	80.4	55.1	55.0	55.0	62.0	70.7	78.3	79.6	78.8	77.3	74.5	52.6	53.7	39.9	34.2	42.7	42.7	52.3	56.1	44.6	54.7	62.2	23.1	36.0		71.1	60.9	60.7	59.4	61.3	60.1	27	SOOV M_ORF
28	62.9	71.6	74.6	54.7	56.9	55.5	64.4	71.8	78.3	78.3	79.8	78.4	74.3	53.6	55.9	43.3	40.2	41.6	43.5	51.9	56.3	39.8	57.4	63.2	36.5	37.2	37.1		57.9	60.5	61.1	60.6	59.2	28	DOBV M_ORF
29	63.2	72.0	78.2	63.9	64.9	63.6	64.4	69.0	75.8	79.1	74.9	78.2	79.4	61.0	64.5	53.5	49.5	53.6	60.4	60.9	66.8	61.0	65.4	50.6	61.5	62.3	62.1	62.0		71.1	67.1	66.0	65.1	29	ANDV M_ORF
30	60.8	71.7	74.0	62.5	62.5	63.5	60.9	68.7	77.2	80.6	79.8	75.5	80.8	63.4	63.8	56.1	40.2	52.9	57.6	60.4	62.6	59.6	65.3	50.9	62.3	63.8	60.2	60.9	36.1		66.9	68.6	67.4	30	SNV M_ORF
31	60.8	70.4	72.3	63.8	63.7	64.3	61.2	69.8	75.0	80.2	80.5	74.7	74.5	63.7	60.3	50.7	46.5	53.8	55.0	62.5	63.1	58.5	62.5	49.5	61.7	61.0	63.2	61.6	46.4	45.4		72.2	70.7	31	PUUV M_ORF
32	60.7	67.1	71.1	61.0	60.7	60.6	61.8	66.4	75.1	76.3	75.9	74.5	72.3	61.2	63.1	50.7	44.3	54.8	58.6	61.1	63.0	58.0	61.7	46.5	59.5	59.4	58.5	61.2	46.0	43.0	35.7		73.3	32	TULV M_ORF
33	63.1	70.2	71.7	65.3	62.1	64.0	60.1	69.0	79.2	79.8	78.4	74.7	77.8	66.8	64.3	54.6	50.3	55.8	59.5	63.4	62.0	66.3	64.3	49.2	61.5	63.3	61.6	64.2	48.9	45.0	37.7	33.2		33	PHV M_ORF
	1	2	3	4	5	6	7	8	9	10	11	12	13	14	15	16	17	18	19	20	21	22	23	24	25	26	27	28	29	30	31	32	33		

L seg pairwise table

MegAlign - [Pair Distances of 230425_ASTV_Lnt_final.meg ClustalW (Slow/Accurate, IUB)]

File Edit Align View Options Net Search Help

Percent Identity

Divergence

	1	2	3	4	5	6	7	8	9	10	11	12	13	14	15	16	17	18	19	20	21	22	23	24	25	26	27	28	29	30	31	32	33	34	35	36	37	38	39	40	41	42	43	44	45	46	47	48	49		
1		39.4	69.9	70.7	64.8	67.7	67.2	69.8	80.5	70.2	69.0	67.5	67.1	47.2	68.3	69.1	66.5	67.6	62.6	66.1	67.2	66.8	67.8	66.0	68.5	69.2	69.2	67.9	68.0	60.0	67.6	68.2	66.5	59.6	67.4	67.9	67.3	67.0	67.1	67.6	68.3	66.0	66.4	67.6	67.7	65.4	66.3	67.8	69.1	1	3877_L1982F_LR2_1369bp
2	49.3		85.8	87.2	3.0	41.8	42.1	52.8	48.3	53.3	42.3	82.4	76.9	70.7	73.6	70.7	71.4	74.9	69.3	71.7	69.8	68.8	73.9	4.5	41.7	71.0	67.2	66.7	43.7	74.1	69.0	71.4	66.2	69.1	65.7	69.4	71.3	69.8	67.1	70.7	67.4	69.4	70.3	66.4	69.8	67.0	66.4	66.4	64.7	2	3873_L2855F_L3855R_693bp
3	40.7	16.4		86.4	4.5	67.0	67.6	67.6	66.5	67.9	69.0	77.8	74.4	69.3	72.4	68.2	66.2	73.3	73.3	69.3	70.2	70.5	70.7	20.5	68.2	67.9	67.0	65.6	65.9	69.3	72.2	67.6	65.6	66.2	66.5	66.2	67.9	65.9	67.9	67.0	69.3	65.3	53.1	67.0	65.6	67.6	65.6	66.2	68.8	3	3931_LF2LR2_352bp
4	39.9	14.6	15.5		12.5	67.0	66.8	66.8	63.6	67.6	67.0	76.4	75.0	69.0	71.0	68.2	65.0	74.7	73.0	67.6	70.7	71.3	68.5	9.4	65.9	65.6	66.2	64.5	64.5	66.8	68.8	66.8	63.9	65.3	64.2	63.4	66.8	61.9	65.6	65.6	67.9	62.2	61.9	66.2	64.5	63.4	63.4	65.6	69.7	4	3943_LF2LR2_352bp
5	44.7	0.0	0.0	0.0		60.1	62.2	16.5	16.8	16.3	61.4	79.3	81.8	25.4	69.2	10.5	13.7	68.7	10.6	69.2	70.9	69.3	69.8	68.9	31.8	68.5	68.3	65.8	64.9	13.8	68.1	69.7	64.3	10.5	68.7	67.1	68.3	66.9	68.4	67.9	67.7	65.9	68.7	65.6	68.4	65.8	66.3	67.0	17.1	5	3945_L1924F_L2900R_977bp
6	44.3	47.4	44.5	45.1	44.[illegible]		90.8	92.0	79.0	90.4	91.5	66.7	67.3	44.7	68.8	63.3	64.7	67.1	56.8	66.8	67.2	68.0	66.3	64.2	68.4	72.0	71.5	71.7	72.0	60.5	71.7	70.8	68.6	62.0	70.3	69.6	68.8	71.2	71.7	70.5	71.4	69.0	66.5	67.7	69.0	67.8	76.8	73.8	72.5	6	3879_L1929F_LR2_1327bp
7	44.0	47.5	44.0	45.7	45.5	10.0		99.6	78.8	89.9	90.4	67.1	68.4	43.9	68.7	62.7	64.1	67.7	56.3	67.7	67.8	68.4	68.4	64.2	66.6	71.4	70.8	72.0	71.4	60.5	72.3	71.5	68.2	62.0	70.0	70.5	68.6	70.2	71.4	70.4	71.6	67.6	66.0	68.4	67.7	67.7	75.1	73.6	74.3	7	3884_L1929F_LR2_1327bp
8	39.9	47.5	44.0	45.7	31.4	8.6	0.4		79.0	89.9	90.2	68.2	69.8	66.7	66.9	62.7	64.1	68.7	56.3	68.9	68.4	68.5	69.3	25.3	70.2	72.9	68.9	72.5	71.1	60.5	73.4	71.1	70.7	62.0	71.2	71.2	71.2	69.4	71.1	70.7	70.5	68.2	65.8	69.4	67.3	67.1	74.9	74.5	74.3	8	3890_L2770F_LR2_553bp
9	24.5	49.5	45.6	51.2	31.4	25.6	25.0	24.8		79.7	79.7	67.5	66.7	67.8	68.5	62.0	63.8	66.2	54.3	68.7	67.1	67.8	68.5	25.7	73.4	71.1	69.4	71.6	72.0	59.6	71.1	71.2	70.3	60.3	69.6	69.3	69.1	66.5	70.9	70.9	72.0	66.0	67.8	70.0	68.9	67.8	71.4	70.7	70.2	9	3914_L2770F_LR2_553bp
10	40.6	46.6	43.5	43.5	38.4	10.5	11.2	11.2	24.5		89.2	68.2	67.5	66.4	67.6	62.0	65.3	69.8	56.3	68.9	69.3	68.9	68.0	25.5	72.7	74.3	73.1	72.7	72.2	61.2	74.9	72.7	71.6	62.3	70.5	71.6	70.9	70.7	70.5	71.2	71.4	69.8	65.8	68.7	68.9	69.3	76.3	75.6	73.4	10	3930_L2770F_LR2_553bp
11	42.3	46.6	41.9	45.7	45.8	9.2	10.5	10.8	24.5	12.0		66.5	67.2	44.4	68.6	61.7	65.6	67.4	55.6	67.8	68.3	68.0	67.2	64.5	68.0	71.5	71.5	72.3	71.4	60.3	72.5	71.1	69.8	60.6	70.5	70.5	68.1	71.1	71.7	71.7	71.9	67.3	66.5	68.4	68.8	67.0	77.1	73.6	74.0	11	3947_L1929F_LR2_1327bp
12	46.0	20.8	26.6	28.6	24.3	47.2	46.1	47.3	47.1	45.9	47.5		85.7	71.0	68.8	67.9	71.7	70.4	68.8	65.9	66.2	64.6	66.7	68.5	69.8	66.3	63.9	65.1	62.8	71.2	64.4	66.5	64.6	70.3	65.6	64.8	65.4	65.7	65.3	65.2	65.0	64.8	65.6	65.3	65.4	64.1	64.3	64.6	65.9	12	NVAV_Te34L_ORF
13	46.0	28.0	31.7	30.7	21.0	46.5	45.3	42.7	49.9	46.5	47.1	16.6		72.6	67.8	70.1	70.6	71.3	70.0	65.8	66.0	65.1	66.1	69.4	69.6	66.4	64.1	65.2	61.7	69.9	64.2	65.3	64.7	67.4	66.2	64.0	65.5	65.0	65.4	64.8	65.2	64.3	65.5	66.0	65.5	63.4	64.5	65.1	64.7	13	NVAV L_ORF
14	40.5	35.2	41.5	44.8	34.4	44.5	47.9	45.8	44.1	44.7	45.4	37.9	35.7		75.4	71.3	67.1	63.1	73.7	69.7	72.2	70.0	71.6	27.8	67.0	70.4	70.3	70.3	45.9	69.9	70.7	68.5	68.5	66.2	69.7	69.6	69.4	70.2	70.3	69.5	70.1	70.9	71.7	70.3	69.2	69.4	68.1	68.1	67.3	14	XSV_2589_L2520F_L3880R_1160bp
15	42.7	35.5	35.4	40.1	40.6	43.0	43.4	46.7	44.1	46.8	43.4	43.3	44.1	30.6		69.8	70.0	71.7	72.2	65.8	66.0	65.7	65.5	70.9	70.7	65.2	65.1	64.6	64.0	71.9	64.8	65.4	65.5	69.1	65.8	65.2	66.0	65.7	65.2	65.3	65.6	65.4	64.8	67.1	66.1	64.8	65.4	65.0	68.5	15	LBV L_ORF
16	37.2	37.3	39.1	39.2	0.0	46.4	47.6	47.6	47.0	48.3	48.9	41.5	39.5	36.3	38.3		56.5	70.4	70.7	69.4	73.8	70.7	70.4	30.2	67.3	68.2	67.9	65.7	65.1	69.8	71.6	63.9	64.5	69.1	68.5	64.2	68.2	64.8	67.9	66.7	66.7	64.8	67.3	69.1	68.5	66.0	65.4	64.8	66.7	16	Longquan_Ra10 L
17	46.4	37.1	37.6	42.1	0.0	45.9	47.0	47.0	48.5	48.5	44.2	35.3	38.3	43.6	40.0	43.6		73.2	68.8	67.1	70.6	67.3	69.1	6.4	67.3	69.4	67.3	66.5	63.6	68.2	67.6	67.3	67.9	64.7	67.1	68.2	68.5	66.2	66.2	66.8	68.5	66.2	63.8	64.1	62.7	64.1	68.2	67.1	65.0	17	Huangpi_Pa1 L
18	46.4	33.1	35.0	32.6	40.6	45.5	44.3	42.0	48.2	41.0	44.2	38.3	37.1	38.9	37.7	38.1	33.7		69.6	70.0	70.7	70.0	71.3	68.8	68.8	69.9	69.7	70.7	56.9	68.8	68.4	68.6	69.1	66.9	70.1	68.6	70.4	67.0	68.9	69.0	68.9	66.7	69.4	68.4	68.1	68.5	68.7	69.0	68.2	18	MOYV KB576_L1691bp
19	39.1	41.6	35.0	36.4	0.0	47.4	47.3	47.3	51.7	50.1	49.6	44.6	42.5	35.0	35.0	39.1	41.5	42.1		69.6	73.4	70.3	70.0	8.2	62.1	69.1	69.6	63.8	60.9	65.9	65.7	66.2	65.0	66.2	66.2	68.1	67.4	63.5	65.5	65.7	65.2	66.4	64.5	66.4	65.5	65.5	65.0	65.7	71.1	19	MGBV_1209_Lseg
20	48.6	38.1	41.2	45.2	40.3	46.7	47.1	46.2	44.0	44.1	45.5	50.3	50.5	38.9	48.6	39.5	45.7	39.3	44.2		74.4	70.1	71.3	73.5	67.4	66.3	64.1	64.7	63.1	70.1	64.3	64.4	65.0	71.8	64.3	64.1	64.9	64.7	64.1	65.2	64.9	65.1	64.7	65.7	64.5	63.7	63.6	64.6	69.4	20	MJNV L_ORF
21	45.1	37.3	43.1	42.5	39.1	46.8	47.4	44.4	45.9	44.0	44.8	49.6	49.4	35.5	49.2	32.4	38.7	39.0	33.2	32.0		69.7	69.3	72.4	69.3	65.2	63.2	63.9	62.8	71.7	63.5	63.8	63.6	71.5	64.1	63.5	63.6	64.7	63.5	64.7	63.8	64.1	64.9	65.3	64.9	63.5	63.9	64.5	71.4	21	TPMV L_ORF
22	46.2	38.3	44.1	42.1	42.0	45.3	44.6	45.8	44.3	43.5	44.0	51.4	50.5	39.3	49.4	37.3	44.0	39.9	39.4	40.1	40.1		71.0	74.5	67.4	67.2	64.9	64.7	62.0	69.0	65.2	64.2	64.6	70.6	65.2	64.1	64.1	64.6	64.9	64.2	64.3	64.6	63.9	65.1	64.2	62.9	64.1	65.0	69.1	22	ULUV L_ORF
23	46.7	34.7	41.2	45.7	40.2	46.7	43.5	40.8	43.4	43.0	44.9	48.7	48.3	37.5	49.0	37.9	41.6	37.9	42.2	37.7	40.7	37.7		75.2	67.8	66.6	64.4	65.6	62.9	74.3	65.1	64.7	66.0	71.3	65.6	65.5	65.5	64.0	64.9	64.5	64.6	64.7	65.0	65.1	64.9	64.0	63.9	64.5	69.9	23	KMJV L_ORF
24	41.7	51.8	0.0	0.0	41.1	43.1	41.7	40.0	35.1	39.9	40.7	42.7	40.4	39.0	38.4	0.0	9.8	42.1	54.9	34.9	35.6	32.3	31.7		36.8	71.0	69.1	69.2	67.8	4.0	66.7	70.2	66.5	10.7	69.5	70.2	71.2	68.5	68.9	67.5	68.2	68.9	69.8	69.8	71.1	66.6	68.6	67.6	7.2	24	DHCV_514_L1900_2970
25	44.9	44.7	44.1	47.0	34.9	41.4	44.0	41.4	35.5	37.5	41.4	40.7	41.6	45.5	39.5	41.1	40.8	42.1	43.3	43.7	41.5	44.6	44.5	42.6		72.6	71.5	71.1	71.7	66.1	71.2	73.3	71.0	64.2	70.7	71.4	68.8	69.3	69.9	69.2	69.8	70.3	68.7	70.6	69.4	67.0	68.7	67.6	74.6	25	BOGV_2074_L2541_3323nt
26	42.7	36.2	43.1	45.7	42.5	35.9	36.3	34.4	38.5	32.4	36.4	49.9	49.2	38.5	49.8	42.1	38.5	39.6	40.5	50.5	51.7	49.1	48.9	40.1	35.7		71.1	74.2	72.3	82.1	75.2	74.8	72.6	73.0	71.6	72.0	68.6	70.4	70.8	70.7	70.4	68.2	68.8	68.5	68.7	66.9	70.8	71.3	73.4	26	ASAV L_ORF
27	43.1	41.7	47.5	49.9	43.1	36.3	37.4	40.4	39.6	34.8	36.0	53.4	53.7	39.6	51.0	41.9	44.3	40.6	38.4	54.5	55.1	52.8	51.8	41.0	38.7	34.6		66.5	71.5	77.5	66.3	72.9	69.6	73.2	68.5	70.5	67.6	68.9	69.6	69.9	68.1	67.5	66.6	67.8	67.7	66.9	69.9	70.6	73.4	27	OXBV L_ORF
28	45.2	40.4	47.4	50.8	48.1	35.9	35.5	34.6	36.4	34.9	35.1	51.8	50.3	38.6	50.8	44.8	45.1	38.6	47.9	52.4	52.8	50.8	49.9	43.2	37.9	32.2	34.5		64.0	76.6	74.9	77.5	71.9	73.2	67.9	72.0	67.7	71.2	71.8	71.5	71.4	67.8	67.6	67.6	68.3	67.1	70.6	71.2	71.1	28	JMSV L_ORF
29	43.3	44.4	48.2	49.8	49.5	36.5	37.9	36.9	36.3	35.1	36.6	58.7	59.1	45.6	55.0	47.0	52.3	44.3	45.8	57.6	58.2	59.7	56.3	43.4	38.5	31.2	37.4	35.8		69.6	68.2	73.5	69.3	65.9	67.3	68.9	66.7	68.8	69.4	68.5	69.4	66.1	66.8	65.0	67.1	66.5	69.0	69.4	73.7	29	SWSV L_ORF
30	37.3	32.5	40.2	43.2	0.0	35.1	35.0	35.0	36.8	34.2	35.5	39.4	42.1	38.7	35.6	39.2	41.1	41.7	44.8	39.7	37.4	41.4	32.5	10.8	28.2	20.6	28.6	29.8	23.9		82.4	78.6	80.1	75.4	75.4	74.8	71.9	75.7	75.4	73.7	74.8	72.8	70.5	71.9	69.4	72.8	71.9	73.2	74.0	30	QHSV L_ORF
31	45.9	35.5	40.0	41.7	45.3	36.7	35.4	33.6	37.6	31.2	35.2	51.7	53.1	37.9	49.8	34.6	41.0	41.3	45.8	52.5	54.5	51.3	50.5	47.6	39.5	30.6	36.4	31.0	27.6	20.4		75.1	71.1	73.5	71.4	71.4	66.7	70.2	69.9	70.1	69.8	66.7	67.8	67.2	66.2	66.9	68.9	70.0	75.1	31	KKMV L_ORF
32	45.4	36.5	45.2	46.3	41.0	38.4	37.5	37.6	37.6	35.7	37.8	49.5	50.0	40.0	48.8	48.4	43.7	43.2	47.0	52.2	53.1	52.4	52.4	40.1	35.3	31.4	35.1	27.3	33.8	25.6	30.8		71.2	72.5	72.2	72.2	68.0	71.6	70.7	71.5	72.0	67.8	68.1	68.5	68.4	67.1	71.1	71.2	71.4	32	CBNV L_ORF
33	47.1	41.4	48.2	48.0	50.4	43.1	43.0	38.2	39.1	36.6	40.2	53.2	52.1	42.8	49.2	49.0	45.8	41.2	47.9	51.2	53.0	52.8	49.9	46.5	39.3	35.5	41.4	35.9	41.7	24.2	38.1	37.1		73.5	72.3	73.7	68.3	70.3	70.0	70.6	70.3	67.5	68.3	67.9	67.6	66.7	69.2	69.6	74.0	33	JJUV L_ORF
34	44.9	44.1	50.4	49.3	0.0	39.2	39.1	39.1	40.6	39.1	41.0	43.2	46.8	49.0	43.4	39.7	46.9	46.0	45.1	36.3	36.7	41.2	40.7	0.0	36.6	35.4	33.4	34.6	35.9	31.3	32.9	36.6	34.3		79.6	76.2	68.6	70.3	69.1	71.5	71.8	70.8	68.4	74.9	68.9	71.0	71.8	69.8	74.9	34	TGNV L_ORF
35	45.4	41.1	47.1	48.8	43.3	40.5	40.5	38.2	40.7	39.2	39.8	51.8	50.0	39.9	50.6	41.9	44.1	39.5	45.1	54.8	54.7	53.3	51.1	43.2	39.5	36.8	38.4	36.9	39.3	33.3	37.0	35.6	34.9	25.1		74.3	67.4	70.0	69.9	69.6	68.8	67.0	67.8	68.2	67.1	66.4	69.1	69.5	70.5	35	AZGV L_ORF
36	46.4	37.9	47.9	49.9	47.3	39.7	38.1	36.7	41.7	37.0	38.5	51.8	52.5	40.5	50.4	48.3	42.5	41.1	44.3	54.2	54.8	53.5	50.8	41.3	38.3	36.4	38.3	36.0	42.0	31.2	37.0	35.9	33.6	29.1	32.7		67.8	69.7	69.1	70.2	69.9	67.2	66.2	67.1	66.5	66.4	68.9	70.1	68.8	36	BOWV L_ORF
37	46.7	37.3	43.0	44.6	43.8	44.2	42.4	38.9	46.3	40.4	42.8	50.1	49.3	40.1	49.1	42.8	41.7	39.7	42.1	50.6	54.9	53.5	49.4	37.6	45.3	43.9	46.6	44.3	49.6	38.2	46.2	44.6	44.9	42.7	45.8	46.0		68.0	68.0	68.0	68.0	71.8	72.2	71.9	71.3	70.9	67.1	67.0	70.8	37	RKPV L_ORF
38	47.9	40.4	51.7	55.8	46.2	38.3	39.6	41.3	46.9	40.5	38.0	51.2	51.6	40.2	50.1	46.0	44.7	45.8	51.1	52.6	52.7	51.6	53.7	44.2	42.1	39.1	41.9	37.7	42.2	29.7	39.1	36.9	40.2	40.7	40.0	40.8	45.5		74.8	81.3	75.0	67.7	68.0	67.9	67.4	66.9	70.2	69.1	70.5	38	HTNV L_ORF
39	48.5	42.4	45.0	50.6	44.6	37.0	37.9	37.4	39.4	37.7	37.0	51.6	50.9	40.2	49.3	41.4	43.5	41.7	47.6	53.5	54.2	52.0	50.1	43.2	39.3	39.1	41.7	37.0	41.3	29.8	40.2	38.0	41.0	41.0	41.5	41.5	45.7	31.9		75.5	75.3	67.4	68.2	68.8	67.5	67.5	70.0	70.6	70.8	39	SEOV L_ORF
40	45.7	38.2	47.5	49.4	45.6	38.9	39.4	38.8	38.6	37.7	36.7	52.7	52.4	40.3	49.7	43.7	46.9	41.1	47.7	50.6	52.6	52.2	52.0	45.5	41.8	39.0	41.1	37.0	43.1	32.8	39.6	36.9	39.6	39.0	41.1	40.0	45.6	21.9	30.7		74.9	68.1	67.8	68.2	68.2	66.6	69.7	69.7	72.5	40	SOOV L_ORF
41	45.7	40.0	42.1	42.0	43.8	36.9	36.4	38.4	36.6	37.1	35.6	51.3	50.4	38.6	49.4	43.2	39.0	41.3	46.7	52.2	53.9	52.6	51.8	43.4	40.7	39.3	42.8	36.6	41.7	33.0	40.2	36.3	40.0	40.2	42.4	39.9	44.8	31.2	30.9	31.2		67.6	68.5	68.5	67.8	67.0	68.9	69.5	73.4	41	DOBV L_ORF
42	49.5	38.7	47.8	52.7	46.7	43.8	46.1	44.2	45.9	40.6	45.5	51.8	52.0	38.1	49.2	48.4	47.7	45.1	49.1	51.5	54.0	51.9	52.0	41.0	41.3	44.4	45.6	45.1	49.1	35.5	45.8	44.6	45.7	40.1	46.5	46.4	36.4	45.5	45.5	45.1	45.2		75.7	72.2	72.2	70.7	66.6	66.8	68.5	42	ANDV L_ORF
43	48.1	38.5	52.1	53.8	42.9	48.2	47.8	50.0	44.5	48.4	46.6	50.6	50.1	37.0	49.2	42.6	50.1	41.3	50.3	52.4	53.3	53.2	50.7	39.1	42.7	43.3	47.1	44.5	49.6	39.2	44.6	44.5	45.0	41.7	45.8	48.5	36.0	46.0	44.8	45.5	44.2	30.1		71.6	71.9	71.0	67.3	67.5	68.8	43	SNV L_ORF
44	44.4	40.6	45.6	44.4	47.7	43.1	42.4	40.9	42.4	41.9	42.9	51.3	49.9	38.4	46.0	42.8	52.2	42.9	45.6	51.2	51.5	51.3	50.6	41.4	41.2	43.8	44.9	44.2	49.8	35.8	46.0	43.4	46.0	30.5	45.3	47.3	36.7	45.4	44.3	44.9	44.4	36.1	37.1		75.6	73.3	67.5	67.3	70.2	44	PUUV L_ORF
45	45.6	39.8	46.5	47.0	45.1	44.5	45.6	45.6	43.9	41.7	43.1	51.2	50.5	41.9	49.1	48.7	49.6	42.2	48.8	52.7	52.2	52.9	51.3	38.6	45.1	43.4	45.0	43.8	48.4	42.0	47.0	44.2	46.2	41.9	46.6	47.4	37.1	46.5	45.6	44.7	45.1	36.0	36.3	30.8		74.6	67.2	67.0	67.3	45	TULV L_ORF
46	49.9	38.9	45.2	51.1	47.8	44.4	44.8	45.0	44.7	42.6	45.5	54.1	53.7	40.5	50.2	51.9	50.5	42.9	51.5	53.0	54.4	55.1	52.9	46.5	48.8	47.2	47.3	46.4	49.1	36.3	47.2	47.3	47.5	39.0	47.7	49.2	38.4	47.3	46.0	48.8	46.9	38.5	38.2	34.3	31.7		66.2	67.2	67.6	46	PHV L_ORF
47	48.2	42.2	46.9	51.0	47.9	28.3	30.6	30.9	36.1	28.6	27.9	53.6	52.8	42.6	49.6	45.5	41.6	41.9	47.8	54.1	53.5	52.7	53.5	43.5	41.3	38.9	40.3	37.9	42.0	35.7	41.2	38.1	41.9	37.5	41.7	42.2	46.7	40.2	40.9	41.5	42.8	47.7	46.2	46.1	46.4	48.6		80.7	70.8	47	BRGV_DE_MF683846_Lorf
48	45.3	42.4	46.8	45.6	45.7	32.6	32.8	31.4	37.4	29.8	32.9	52.9	52.2	43.7	50.1	50.3	44.9	41.1	45.3	52.7	53.8	51.7	52.5	46.5	43.4	37.9	39.7	38.0	40.7	34.4	38.5	38.0	41.4	39.8	40.3	40.2	46.5	42.1	39.7	41.4	41.6	47.2	45.4	46.1	46.5	46.8	22.7		70.8	48	BRGV_BE_KX551962_Lorf
49	44.0	48.4	42.5	41.9	0.0	34.5	31.7	31.7	38.2	34.1	32.1	48.4	50.8	44.6	43.5	41.5	42.0	47.7	38.6	41.2	38.8	42.3	40.1	0.0	31.4	34.2	35.5	37.4	35.9	33.2	30.5	36.5	33.7	32.2	38.0	41.6	39.2	39.6	37.5	34.9	33.3	43.2	40.9	42.0	47.9	48.0	37.6	37.5		49	Academ virus_Ta450_346bp_L
	1	2	3	4	5	6	7	8	9	10	11	12	13	14	15	16	17	18	19	20	21	22	23	24	25	26	27	28	29	30	31	32	33	34	35	36	37	38	39	40	41	42	43	44	45	46	47	48	49		
